# Anti-biofilm Approach in Infective Endocarditis Exposes New Treatment Strategies for Improved Outcome

**DOI:** 10.3389/fcell.2021.643335

**Published:** 2021-06-18

**Authors:** Christian Johann Lerche, Franziska Schwartz, Marie Theut, Emil Loldrup Fosbøl, Kasper Iversen, Henning Bundgaard, Niels Høiby, Claus Moser

**Affiliations:** ^1^Department of Clinical Microbiology, Rigshospitalet, Copenhagen University Hospital, Copenhagen, Denmark; ^2^Department of Cardiology, Rigshospitalet, Copenhagen University Hospital, Copenhagen, Denmark; ^3^Department of Cardiology, Herlev and Gentofte Hospital, University of Copenhagen, Herlev, Denmark; ^4^Department of Emergency Medicine, Herlev and Gentofte Hospital, University of Copenhagen, Herlev, Denmark; ^5^Costerton Biofilm Center, Department of Immunology and Microbiology, University of Copenhagen, Copenhagen, Denmark

**Keywords:** biofilm, infective endocarditis, innate immunity, *Staphylococcus aureus*, dabigatran, hyperbaric oxygen therapy, *in vivo*, *in vitro*

## Abstract

Infective endocarditis (IE) is a life-threatening infective disease with increasing incidence worldwide. From early on, in the antibiotic era, it was recognized that high-dose and long-term antibiotic therapy was correlated to improved outcome. In addition, for several of the common microbial IE etiologies, the use of combination antibiotic therapy further improves outcome. IE vegetations on affected heart valves from patients and experimental animal models resemble biofilm infections. Besides the recalcitrant nature of IE, the microorganisms often present in an aggregated form, and gradients of bacterial activity in the vegetations can be observed. Even after appropriate antibiotic therapy, such microbial formations can often be identified in surgically removed, infected heart valves. Therefore, persistent or recurrent cases of IE, after apparent initial infection control, can be related to biofilm formation in the heart valve vegetations. On this background, the present review will describe potentially novel non-antibiotic, antimicrobial approaches in IE, with special focus on anti-thrombotic strategies and hyperbaric oxygen therapy targeting the biofilm formation of the infected heart valves caused by *Staphylococcus aureus*. The format is translational from preclinical models to actual clinical treatment strategies.

## Introduction

Infective endocarditis (IE) is defined as an infection of the inner surface of the heart, the endocardium, most prevalent on the heart valves, or on implanted cardiac devices (Moreillon and Que, 2004). In most cases, left-sided IE is considered a more complicated and severe infection compared with patients affected by right-sided IE. Although IE is relatively rare, it is increasing in incidence based on quality reports mainly from high income countries (Holland et al., 2017; Jensen et al., 2020). IE is one of the most serious infections with 100% mortality if untreated and a high rate of complications. Under modern treatment, especially if infectious control is not obtained and septic shock develops, a mortality rate of 20–25% (Murdoch et al., 2009) and a 1-year mortality rate of up to 40% are reported (Alestig et al., 2000; Cabell et al., 2002; Que and Moreillon, 2011). The highly critical appearance of the infections indicates a need for improvement in diagnosis and treatment strategies (Fowler et al., 2005; Que and Moreillon, 2011; Werdan et al., 2014). Rheumatic heart disease and IE in younger persons are still dominant features in low-income countries (Werdan et al., 2014). In contrast, an increase in the population of elderly people is reported from most high-income countries, resulting in further predisposed persons for IE (Werdan et al., 2014). Modern medical treatment also seems to predispose for IE, not the least cardiac implants, hemodialysis, and additional reasons for intravascular catheters (i.e., of cancer and short bowel disease patients), including other patients in risk of healthcare-associated infections due to invasive procedures (Tong et al., 2015). Especially cardiac implants together with improved diagnosis and awareness of IE seem to drive this development. Importantly, an increasing age of the affected population also leads to challenges in therapy due to reduced organ functions, increased susceptibility to potential antibiotic toxicity, and comorbidities.

**FIGURE 1:**
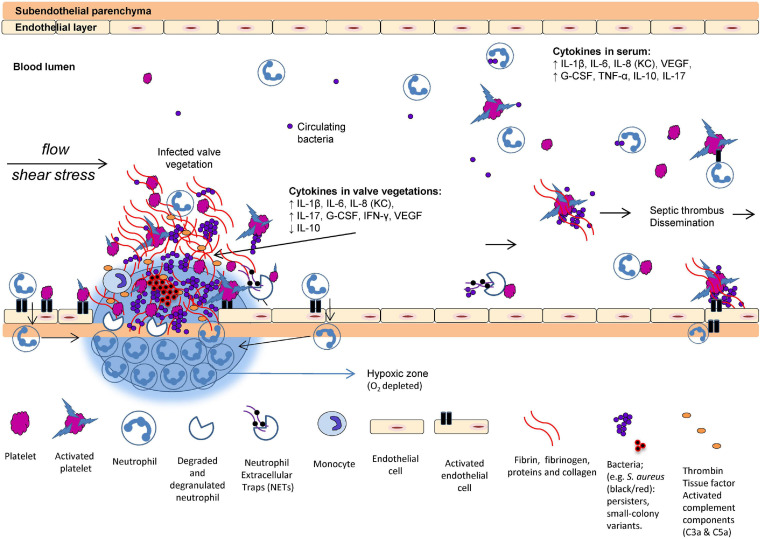
Proposed scenario of interaction between the host and pathogens in infective endocarditis (IE). The formation of valve vegetations is induced by the damaged and infected endothelium. Platelets aggregate to the injured endothelial cells followed by accumulation of innate immune cells and upregulation of tissue factor, fibrinogen, fibrin, and cytokines. O_2_ consumption in the valve vegetations (biofilm) and by the activated neutrophils may prevent appropriate O_2_ in the tissue. Increased IL-8, interaction between activated platelets and neutrophils trigger the formation of neutrophil extracellular traps (NETs). Many bacteria exhibit virulence mechanisms to survive the NET formation. Deep-seated bacteria are less metabolically active, consequently reducing the efficacy of oxygen-dependent antibiotics. In the local tissue of the valve vegetations, key inflammatory markers of progression are elevated, for example, G-CSF, IL-1β, IL-6, IL-8 (analog to KC in rodents), IL-17, IFN-γ, and VEGF (Moser et al., 2017) (with permission from the editor of APMIS and authors).

In geographical areas with quality data, often high-income countries, *Staphylococcus aureus* on native heart valves and coagulase negative staphylococci (CoNS) on implants, is referred to as the dominant IE etiology, closely followed by viridans streptococci (i.e., *Streptococcus mutans*, *S. sanguis*, *S. sanguinis*, *S. mitis*, *S. salivarius*, and *S. bovis*) (Lalani et al., 2006; Murdoch et al., 2009; Østergaard et al., 2019). The third most common bacterial etiology causing IE on native heart valves is *Enterococcus faecalis*, especially in patients above 70 years of age (Hoen and Duval, 2013; Østergaard et al., 2019). These three groups of Gram-positive bacteria constitute approximately 80% of all microbial etiologies of IE (Que and Moreillon, 2011; Østergaard et al., 2019). Substantially less frequent are species of the HACEK group [*Aggregatibacter* (formerly *Haemophilus*) *aphrophilus/paraaphrophis*, *Aggregatibacter actinomycetemcomitans*, *Cardiobacterium hominis*, *Eikenella corrodens*, and *Kingella* spp.] identified as the fourth most common cause of IE on native heart valves (Que and Moreillon, 2011). Indeed, the intact endocardium, including the heart valves, is resistant to microbial colonization (Werdan et al., 2014). However, if micro-lesions of the endocardium appear, a healing process involving fibrin formation and thrombocyte aggregation is initiated with the formation of non-bacterial thrombotic endocarditis (NBTE) (Figure 1; Werdan et al., 2014). The healing process of the endothelium is estimated to last for 2 weeks, and in this period, the breakage of the endothelial barrier is highly susceptible to bacterial colonization (Werdan et al., 2014). The bacteria will adhere to the damaged heart valve by means of surface adhesins, consisting of proteins and polysaccharides (reviewed by Que and Moreillon, 2011). Such colonization can occur during temporary bacteremia of less than 10 min duration due to activities potentially resulting in minor lesions in the oral cavity, like tooth brushing, additional mucosal surfaces lesions, or from the skin microbiota (Hall et al., 1999; Werdan et al., 2014). An established infection subsequently induces an inflammatory response, with recruitment of polymorphonuclear neutrophils (PMNs), resulting in further damages of the heart valve, thus, worsening the process (Werdan et al., 2014).

The following biofilm IE review is divided into sections on *Early host–pathogen interactions*, *Clinical biofilms*, *IE biofilm characteristics and appearance*, *Antibiotic tolerance*, *Small colony variants*, *In vitro and in vivo model systems*, and *Potential novel IE treatments*.

### Early Host–Pathogen Interactions in Infective Endocarditis

The first essential step in the development of IE is the interaction of the pathogen and the activated endothelial layer and platelets (Figure 1). Several adhesive molecules are expressed on the cell wall of Gram-positive bacteria and facilitate the bridging and adherence to the endothelial cells, platelets, and the extracellular matrix [fibrin(ogen) and collagen]. These adhesins can either be covalently bound to the cell wall [microbial surface components reorganizing adhesive matrix aolecules (MSCRAMMs)] (Foster and Höök, 1998) or secreted [secretable expanded repertoire adhesive molecules (SERAMs)] (Chavakis et al., 2005). It is well known that these adhesins play an important role in the development and pathogenesis of IE. Most Gram-positive bacteria can bind directly to fibrin(ogen) (Que et al., 2001, 2005; Entenza et al., 2005) as a bridging molecule (Cheung et al., 1991), fibronectin (Que et al., 2005), collagen (Hienz et al., 1996), and integrins of platelets (Siboo et al., 2001; Niemann et al., 2004; Loughman et al., 2005; Veloso et al., 2013) and endothelial cells (Edwards et al., 2012; Pappelbaum et al., 2013; Liesenborghs et al., 2016). These important host–pathogen interactions are thoroughly reviewed elsewhere (Moreillon et al., 2002; Moreillon and Que, 2004; Que and Moreillon, 2011; Werdan et al., 2014).

**FIGURE 2:**
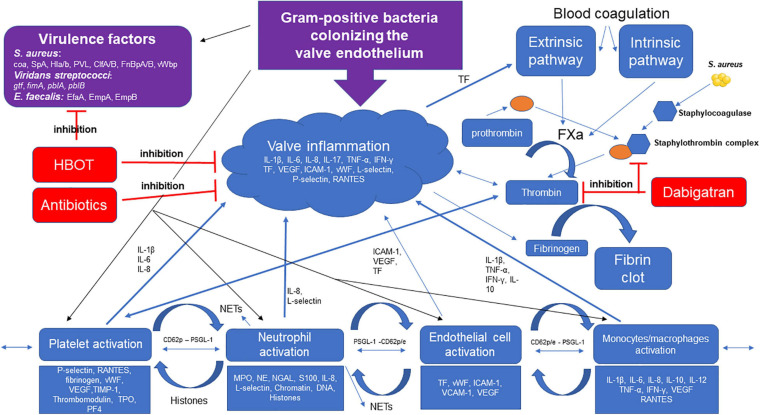
Gram-positive bacteria colonizing the valve endothelium trigger multiple pathways of inflammation. *S. aureus*, *viridans streptococci*, and *E. faecalis* express important adhesion surface proteins/glucans, i.e., clumping factor A and B (ClfA/B), fibrinogen binding protein A and B (FnBpA/B), Coagulase (*coa*) von willebrand binding protein (vWbp), and glucosyltransferase (*gtf*), binding to the activated endothelial cell and extracellular matrix proteins in the vegetation. Host cells (platelets, neutrophils, and monocytes/macrophages) are recruited to the activated endothelium promoted by the pathogen’s increasing production of inflammatory markers. Tissue factor (TF) stimulates the extrinsic pathway increasing thrombin generation converting fibrinogen to fibrin inducing clot formation. Dabigatran is a direct thrombin inhibitor, limiting fibrin formation, HBOT potentiates the effect of antibiotics by decreasing inflammation and decreasing virulence of *S. aureus*. *S. aureus* and thrombin are potent activators of platelets, facilitating recruitment of neutrophil-binding to the surface of the vegetation, stimulating degranulation and neutrophil extracellular traps (NETs) formation, and further enhancing the clot and fibrin formation. The activated endothelial cells also stimulate neutrophil-adhesion and NET release. *S. aureus* produces Staphylocoagulase-forming Staphylothrombin complexes with prothrombin facilitating fibrin formation, dabigatran inhibits the formation of Staphylothrombin complex and fibrin. Abbreviations: *S. aureus*, *Staphylococcus aureus*; *Enterococcus faecalis, E. faecalis*; SpA, *Staphylococcus aureus* protein A; Hl,a/b, alpha and beta hemolysis; PVL, Panton–Valentine leucocidin; *E. faecalis* antigen A, EfA; Interleukin-1beta (-1β, 6, 8, 10, 17); tumor necrosis factor alpha (TNF-α); interferon gamma (IFN-γ); vascular endothelial growth factor, VEGF; tissue factor, TF; intercellular adhesion molecule 1 (ICAM-1); vascular cell adhesion molecule 1 VCAM-1; von Willebrand factor, vWF; Thrombopoietin, TPO; platelet factor 4, PF4; myeloperoxidase, MPO; neutrophil elastase, NE; neutrophil gelatinase-associated lipocalin, NGAL; P-selectin high-affinity ligand, PSGL-1.

Platelets are short-lived cells—they die after approximately 10 days—and is the smallest of the formed blood elements. Platelets were generally thought to be important in hemostatic functions, but are also crucial innate effector cells cross-talking with other innate immune cells and enhancing their functions and recruitment (Yeaman, 1997; Zarbock et al., 2007; Maugeri et al., 2014). Platelets are circulating sensor cells and respond to the activated or damaged endothelium by accumulation of facilitating tissue factor and cytokine production as an initial inflammatory response. Tissue factors are essential for the development of thrombi (von Brühl et al., 2012) and stimulate the thrombin generation, which facilitates the fibrin formation (Panizzi et al., 2011). Activated platelets also release procoagulant molecules facilitating additional platelet aggregation (Rivera et al., 2009). However, these essential and protective properties of the hemostatic system are dysregulated in severe infections and further enhanced by virulent Gram-positive bacteria colonizing the valve endothelium facilitating hyperactivation of complex pathways, resulting in vegetational growth and biofilm formation in cardiac valves (Figure 2).

### Clinical Biofilms

Microbial biofilms can be defined as bacterial aggregates embedded in a self-produced extracellular polymeric matrix constituted of extracellular polysaccharides, proteins, extracellular DNA (eDNA), LPS, and other bacterial products and occasionally also host factors and elements from the surroundings (Høiby et al., 2015). Biofilm research is dominated by thorough investigations on only a limited number of pathogens and clinical syndromes. Therefore, precautions should be taken when transferring results of biofilm infections in general to a specific syndrome, even though there are several common biofilm features. The clinical consequences of biofilm infections are frequent antibiotic treatment failure and relapse of infection when antibiotic therapy is terminated, which cannot be attributed to development of antibiotic resistance *per se* (Høiby et al., 2015). The recalcitrant nature of the biofilm infections has been supported by observations using *in vitro* and *in vivo* animal model systems revealing tolerance of the biofilm-associated microorganisms to both, antimicrobials and the immune system (Høiby et al., 2015). Biofilm growth is probably the preferred lifestyle of extracellular bacteria and yeast in nature and can be considered an ancient survival mechanism (Allen, 2016; Moser et al., 2017). The aggregating microorganisms in the matrix also allow for interbacterial communication, known as quorum sensing—a chemical and bacterial density-dependent mutual impact of the microorganisms on the expression of virulence factors in the microenvironment of the biofilms (Høiby et al., 2010).

### Biofilm Formation in Infective Endocarditis

Whereas it seems well-accepted that device-associated IE involves microbial biofilm formation on the implanted foreign body, biofilm involvement in IE on native valves is more debatable (Marrie et al., 1982). From early observations of penicillin therapy against IE, it became evident that increased and frequent dosing for long treatment periods were necessary for successful outcome, compared with acute infections, indicating a bacterial mode of growth that resulted in a certain tolerance toward the antibiotic provided (Degraff, 1941; Bloomfield et al., 1945; Christie, 1946; Tan et al., 1971). When the bacteria were cultured from the blood or the heart valves at relapse, they still appeared fully penicillin susceptible, when tested *in vitro*, and a second treatment with penicillin could be successful (Christie, 1946; Tan et al., 1971). Relatively early on in the antibiotic era of IE treatments, a synergistic effect of additional streptomycin (and later newer aminoglycosides) to the penicillin therapy against enterococci IE was observed (Hunter, 1947). This approach successfully expanded to the treatment of less penicillin susceptible streptococci. It was also revealed that the synergistic effect was due to inhibition of the cell wall synthesis, allowing intra-bacterial entry of the aminoglycosides (Moellering and Weinberg, 1971).

The recalcitrant nature of IE which necessitates involvement of heart valve surgery in almost 50% of all IE cases, strongly supports the biofilm behavior of IE (Elgharably et al., 2016, 2018). Although, a proportion of surgery in IE is due to valve damage, a substantial fraction of all heart valve surgery during IE is due to insufficient antibiotic effect and lack of infection control (Elgharably et al., 2016, 2018).

Quantification of the bacterial level during IE has revealed a relatively constant level of bacteria in the peripheral blood stream during the natural course of IE (Werner et al., 1967). Performing consecutive blood cultures of patients with streptococcal or staphylococcal IE revealed a substantial varied interpatient level of quantitative bacteriology from less than 10 CFU/ml to above 300 CFU/ml peripheral blood, with a median around 30 CFU/ml (approximately a total of 150,000 bacteria in a person with 5 L of blood) (Werner et al., 1967). However, the individual patient showed a steady level of bacteremia over 2–9 days of observation (Werner et al., 1967). From the onset of appropriate antibiotic treatment, it is reported that the planktonic bacteria of the peripheral blood stream are normally cleared within 48 h, paralleled by a reduction in the valve bacteriology (Bloomfield et al., 1945). However, the free-floating bacteria in the blood stream only constitute an extreme minority of the total IE bacteriology, since the number of CFUs in heart valve vegetations has been reported to be as high as 10^9^–10^11^ CFU per gram tissue (Bennett et al., 2014). This means that the planktonic peripheral blood bacteria at an average only constitute 0.15‰ of the total bacteriology of an IE patient, if we estimate the weight of a vegetation to 0.1 g, and even 0.015‰ in case of a more realistic 1 g vegetation. The majority of the microorganisms are not immediately available for investigations like antibiotic susceptibility testing.

The pronounced chronicity of biofilm infections results in a special immunological dynamic with ongoing activation of both the innate and the adaptive immune responses (Jensen et al., 2010; Moser et al., 2017). This has been investigated thoroughly in patients with the inherited disease cystic fibrosis (CF), suffering from chronic *Pseudomonas aeruginosa* biofilm lung infections (Høiby et al., 1977; Bjarnsholt et al., 2009). Instead of clearing the chronic lung infection, activation of the adaptive immune response contributes to the pathology by means of forming immune complexes, inducing PMN-dominated inflammation and activating the complement system resulting in collateral tissue damage (Høiby et al., 1977; Jensen et al., 2010; Moser et al., 2017). A parallel can be drawn to patients with IE. Indeed, a specific adaptive humoral immune response has been described in subacute IE by means of precipitating antibodies (Kjerulf et al., 1993). Several immunological phenomena during IE have been recognized, including erythema nodosum, glomerulonephritis, and vasculitis, like Osler’s nodes. During IE, hyperglobulinemia has been described (Laxdal et al., 1968). The presence of IgM anti-IgG rheumatoid factor has been reported in half the patients if IE lasts for more than 6 weeks (Sheagren et al., 1976). Additionally, antinuclear antibodies have also been reported during IE (Bacon et al., 1974). How these auto-antibodies contribute to pathology has not been clarified. Circulating immune complexes have been observed in almost all IE patients (Bayer et al., 1976; Holland et al., 2017). Circulating immune complexes are believed to be involved in the peripheral manifestations of IE, like Osler’s nodes and Roth spots resembling Arthus reaction. Whether the humoral response directly contributes to the pathology is not clarified, although antibodies probably both bind and neutralize bacterial virulence factors beneficially, but also precipitate inflammation and thereby contribute to the pathology. However, therapeutic failure has been demonstrated to correlate with a rise in circulating immune complexes, similar to CF (Kauffmann et al., 1981).

### Infective Endocarditis and Biofilms—Valve Vegetation Appearance

Histopathological evaluations of surgically removed heart valves from IE patients, as well as of heart valves from experimental IE animal studies, has shown that the bacteria are organized in biofilm characteristic microcolony-aggregates within the vegetations (Allen, 1939, 1941; Angrist et al., 1960). In a study on the ultrastructure of six aortic valves, myriads of bacteria were observed in areas where the surface of the vegetation was broken (Marrie et al., 1987). Transmission electron microscopy revealed that the bacteria were embedded in an electron dense matrix, even with negative culture results, and cell wall and cell division appeared abnormal (Marrie et al., 1987). Masses of bacteria seen in the vegetations, usually clustered in discrete colonies, were reported many years ago by Allen and colleagues (Allen, 1939). In addition, Hobby et al. (1942), based on histopathological examination of vegetations, suggested that a proportion of the bacteria in the vegetation, though alive and capable of multiplication, are in a resting phase, and hence not subject to rapid killing when exposed to penicillin. Based on observations made in a streptococcal IE rabbit model, Durack and Beeson (1972b) reported gradients of bacterial activity in vegetations, since surface colonies incorporated a Tritium-labeled L-alanin, while deeply seated colonies remained unlabeled, and furthermore, they described that the colonies were surrounded by an amorphous debris (Table 1; Durack and Beeson, 1972b). In a large retrospective study using Gram staining, culture, and histopathology on 506 heart valves removed from patients with IE, a microscopic positive Gram staining of visual bacteria was reported in the majority of the samples (Morris et al., 2003). Even after completed antibiotic treatment, more than 60% of the valves were microscopy positive, increasing to 88% if less than 25% of the treatment was completed (Morris et al., 2003). However, although the majority of the removed heart valves were microscopy positive by means of Gram stain, only 5% were culture positive at completion of antibiotic therapy (Morris et al., 2003). The findings were supported by revealing signs of acute inflammation in approximately one-third of the samples after completion of antibiotic therapy (Morris et al., 2003). By means of fluorescence *in situ* hybridization (FISH) with bacteria-specific probes, Mallmann et al. (2010) revealed causative pathogens in heart valves from patients with culture-negative IE. The authors demonstrated large colonies of streptococci, organized in typical dense bacterial clusters (Mallmann et al., 2010). Additional IE pathogens have also been demonstrated using the FISH technique (Frickmann et al., 2017). These early microscopic descriptions resemble modern definitions and descriptions of biofilms, in accordance with our own clinical and preclinical observations (Table 1; Høiby et al., 2015).

**TABLE 1:** Biofilm characteristics in infective endocarditis (IE).

Description	Pathogen(s)	Clinical study or experimental model	References
Micro-colonies embedded in extracellular matrix components in valve vegetations	α-Haemolytic Streptococci	Rabbit IE model Human autopsy study	Durack and Beeson, 1972a, b; Durack et al., 1973; Durack, 1974; Fernández Guerrero et al., 2012
High density of bacteria per gram tissue and biofilm formation	*S. aureus* *E. faecalis*	Rabbit and rat model of IE	Durack and Beeson, 1972a, b; Durack et al., 1973; Durack, 1974; Bennett et al., 2014; Haller et al., 2014; Lerche et al., 2015
Inoculum effect	*S. aureus*	*In vitro*	LaPlante and Rybak, 2004
Prolonged need for high dosing and combination treatment		Human studies *In vivo* model of IE *In vitro* model of IE	Sande and Courtney, 1976; McGrath et al., 1994; Francioli et al., 1995; Olaison and Schadewitz, 2002; Gavaldá et al., 2003; Dahl et al., 2013; Fernández-Hidalgo et al., 2013
Antibiotic tolerance	*S. aureus*	Systemic *S. aureus* infection in mice	Rowe et al., 2020
Combination treatment improves outcome	Various Gram-positive bacteria	RCT, Partial oral treatment of IE Clinical high-dose trimethoprim-sulfamethoxazole and clindamycin for *S. aureus* IE	Bundgaard et al., 2019; Iversen et al., 2019; Tissot-Dupont et al., 2019
Occurrence of small-colony variants	*S. aureus* *S. epidermidis*	Rat model of IE	Baddour et al., 1988; Bhattacharyya et al., 2012
Oxygen depletion and hypoxia in inflamed valves	*S. aureus*	Pig valves (mitral and aortic) Rat model of IE	Sapp et al., 2016; Lerche et al., 2017; Moser et al., 2017
Foreign body devices or prosthetic valves		Clinical sonication of valves Guinea pig subdermal model Rat model of long-term catheter *In vitro* IE model	Illingworth et al., 1998; Chauhan et al., 2012; Gomes et al., 2018
Platelets, neutrophils contribute to biofilm formation	*Streptococcus* spp., *S. aureus*	Rabbit and rat models of IE	Scheld et al., 1978; Jung et al., 2012; Hsu et al., 2019; Lerche et al., 2019

*S. aureus, Staphylococcus aureus; S. epidermidis, Staphylococcus epidermidis; E. faecalis, Enterococcus faecalis; IE, infective endocarditis; HBOT, hyperbaric oxygen therapy; RCT, randomized clinical trial.*

### Infective Endocarditis and Biofilms—Antibiotic Tolerance

It is known for investigated bacteria and fungi that the MIC—or rather, the minimum biofilm inhibitory concentration (MBIC)—is increased 10- to 1,000-fold for microorganisms in the biofilm mode compared with the exact same microorganism growing planktonically (Anwar et al., 1990; Moskowitz et al., 2004; Hengzhuang et al., 2011). This difference is accentuated if the minimum bactericidal concentration (MBC) is compared with the minimum biofilm eradication concentration (MBEC) (Hengzhuang et al., 2011). In the latter case, it is almost impossible to obtain sufficient antibiotic levels by systemic administration of antibiotics (Hengzhuang et al., 2011). The observed phenomenon is due to the biofilm mode of growth and not mutational antibiotic resistance per se (Fisher et al., 2017). Recently, a study investigating bacterial response to biofilm growing bacteria from a smaller number of IE patients demonstrated how the normal antibiotic susceptibility testing differed from the susceptibility of the bacteria growing as biofilms in an *in vitro* biofilm setup (Di Domenico et al., 2019). The explanation for this biofilm antibiotic tolerance phenomenon is multifactorial (Ciofu et al., 2017). A key reason is believed to be the significantly changed physiology of the biofilms, with gradients from the biofilm surface to the center (Jensen et al., 2017). Inside the biofilm there can be limited access to nutrients and not the least, oxygen (Zhang et al., 2013; Jensen et al., 2017). Indeed, hypoxic conditions just 50 μm inside biofilms have been described (Zhang et al., 2013; Jensen et al., 2017). The result is an adaptation of the bacterial metabolism to the biofilm environment, leading to a significantly reduced activity and prolonged generation time (Kragh et al., 2016). These observations are in concordance with the described microscopic observations of heart valves from patients with IE and from animal models (Allen, 1939, 1941; Angrist and Oka, 1963; Durack and Beeson, 1972a, b; Durack et al., 1973; Durack, 1974). Prolonged division times result in reduced activity of antibiotics, which are dependent on division of the bacterial cells (Tuomanen et al., 1986). The biofilm-generated hypoxia may be adding to the heart valve disease progression as indicated in a study on effect of hypoxia on pathological changes of porcine aortic and mitral valves using an oxygen diffusion chamber (Sapp et al., 2016). Interestingly, vascular endothelial growth factor (VEGF) receptor 2 seemed to be involved in this effect, and we have shown that HBOT was able to reduce VEGF expression levels in the infected aortic valves using a rat model of left-sided *S. aureus* IE (Lerche et al., 2017).

A further consequence of the limited oxygen below the biofilm surface is a reduction in the oxygen-dependent effect of bactericidal antibiotic drugs (Kohanski et al., 2007). Antibiotic exposure to bacteria results in metabolic changes of the bacteria as a stress response, resulting in intra-bacterial accumulation of toxic oxygen radicals, which will cause damage to the DNA/RNA, proteins, cell membranes, and other essential macro-molecules and subsequentially death of the bacteria (Dwyer et al., 2015). This mechanism adds to the target-specific effect of the bactericidal antibiotics, including interactions with cell wall synthesis, DNA replication, or protein synthesis (Jensen et al., 2014). This important oxygen-dependent potentiation of bactericidal antibiotics can be a target to augment the antibiotic efficacy in IE by supplying excessive oxygen under pressure (more details in the *Adjunctive treatment with dabigatran* section).

The efficiency of antibiotic penetration into deep-seeded biofilms and into bacterial vegetations in IE is a topic of extensive research. It may be argued that the antibiotic penetration is lowered by the limited direct blood supply of the valves, the dense valve vegetations, mainly constructed of fibrin and platelets, as well as the bacterial aggregates with their polysaccharide matrix (Elgharably et al., 2018). However, for the heart valve vegetations, antibiotic penetration is probably more refined and individual, and due to the limited access to samples, not thoroughly examined. However, using autoradiographic distribution techniques with labeled antibiotic compounds and experimental animal models, three different diffusion patterns have been described and reviewed by Cremieux and Carbon (1992). The first pattern was observed for teicoplanin with a high concentration of the antibiotic in the periphery of the vegetation, but a limited penetration into the vegetation resulting in decreased anti-bacterial activity (Cremieux and Carbon, 1992). The second pattern was observed for ceftriaxone, where a progressive diffusion, but also a substantial gradient from the vegetation surface to the center was reported (Cremieux and Carbon, 1992). The same pattern, although to a lesser degree, was observed for penicillin. In contrast, a third pattern of diffusion was observed for some fluoroquinolones and tobramycin which provided a homogenous distribution throughout the vegetation (Cremieux and Carbon, 1992). In support, aminoglycosides have, in an experimental study by means of autoradiography, been shown to distribute homogeneously inside the vegetations (Table 1; Cremieux et al., 1989).

The limited antibiotic penetration into the bacterial vegetation in IE can, at least partially, be translated to other well-known biofilm infections characterized by bacterial aggregates surrounded by a polysaccharide matrix. It has been suggested and shown that bacterial biofilms can be considered as independent pharmacokinetic microcompartments (Zimmerli and Moser, 2012; Cao et al., 2015). It was revealed that tobramycin was bound to substances of the biofilm matrix and, thus, not free for bacterial killing (Cao et al., 2015, 2016). The substantial matrix binding resulted in a delayed diffusion of tobramycin following a power law, instead of a First-order kinetic (Cao et al., 2016). Recently, this phenomenon was also shown *in vivo* (Christophersen et al., 2020). Finally, it has been shown that the pharmacokinetics and pharmacodynamics of antibiotics in biofilms follow the same rules (time-, concentration-, or dose-dependent killing) as for planktonically growing bacteria, although significantly higher antibiotic concentrations are needed (Hengzhuang et al., 2011).

The density of the bacterial aggregates and the exceptionally high number of bacteria inside the vegetations also lead to an inoculum effect of the bacteria against different antibiotics (Song et al., 2018). The inoculum effect describes the phenomenon that a high number of bacteria (high inoculum) leads to an increase in MICs, to values even above the breakpoint. This has been reported for β-lactam antibiotics like piperacillin/tazobactam, ampicillin/sulbactam, and, to some degree, oxacillin, whereas cephalosporins and meropenem were less influenced by the inoculum effect (Song et al., 2019). The inoculum effect is likely to also contribute to the reduced antibiotic effect in case of IE.

### Infective Endocarditis and Biofilms—Small Colony Variants

Chronic antibiotic-refractory infections can lead to the appearance of small colony variants (SCV) (Proctor et al., 2006). SCVs obtain a survival advantage by slow and/or intracellular replication and, thereby, reduced susceptibility to antibiotics (Vulin et al., 2018). As the SCVs phenotype is infrequent and most of the times not a consequence of mutations, and therefore not stable, SCVs are often missed in the standard clinical laboratory testing unless there is specific focus on the variants (Table 1; Tuchscherr et al., 2011). For *S. aureus* and IE, SCVs have been intensively studied and reviewed elsewhere, but are characterized as non-hemolytic and non-pigmented colony morphologies 5–10 times smaller than the most common colony morphologies, due to auxotrophy (Proctor et al., 2006; Garcia et al., 2013). On animal experiments, *S. aureus* SCVs appeared from the heart valves of rats with aortic IE treated with tobramycin (Lerche et al., 2017). Interestingly, hyperbaric oxygen therapy (HBOT) significantly increased the appearance of SCVs (Lerche et al., 2017). This could be explained by the increased oxidative stress from HBOT and aminoglycoside treatment, highlighting the need to treat IE with antibiotics, which do not have an oxygen-dependent killing mechanism (i.e., rifampicin and linezolid) to prevent SCVs within the biofilm vegetations. For *E. faecalis*, SCVs have only been sparsely and casuistically described, although in relation to IE (Wellinghausen et al., 2009; Ogihara et al., 2016). However, for *E. faecalis* SCV, reduced sugar fermentation, changed ultrastructural morphology, and growth behavior, as well as changed antimicrobial susceptibility were observed (Wellinghausen et al., 2009). Especially, a reduced aminoglycoside susceptibility was reported (Wellinghausen et al., 2009; Ogihara et al., 2016). Our own observation is that *E. faecalis* SCVs are only identified from surgically removed heart valves of IE patients, and not from the corresponding blood cultures, even though we have had focus on this issue. For streptococci, SCVs have only been sparsely investigated and almost exclusively for *S. pneumoniae*.

## Model Systems of Infective Endocarditis

Experimental representative model systems of IE are essential to identify potentially novel treatment strategies. The recently published POET study is a rare exception of a large randomized controlled trial within IE (Iversen et al., 2019). The conduction of clinical trials in IE is especially challenging because of factors like co-morbidities, different microbiological etiologies, difficulties and delays of diagnosis, the admission of the patients to many different hospitals, as well as surgery in almost half the patients (Bundgaard et al., 2019; Iversen et al., 2019). Therefore, we are dependent on clinically relevant animal models to provide solid preclinical testing of potential improvements for IE. By regarding IE as a biofilm-related infection, new *in vitro* model systems could be developed taking these important aspects into consideration. The benefit of such design could improve screening of new treatment options before heading to preclinical studies and helps in reducing the substantial gap from *in vitro* to *in vivo* and lastly into clinical studies.

### *In vitro* and *ex vivo* Model Systems of Infective Endocarditis

Even though biofilms can have a heterogenic and complex appearance, they share certain characteristics, such as the extracellular polymeric matrix, high cell densities, and decreased susceptibility toward antimicrobial treatments. It is therefore possible to mimic biofilm environments *in vitro* to a certain degree, to improve our understanding of biofilms, and gain insight into possible new treatment options. Multiple different model systems have been developed to study different angles of biofilms. For initial screening or high throughput experiments, *in vitro* models can be exceptionally helpful in identifying lead candidates for novel treatment strategies to be tested in more clinically relevant model systems. However, the majority of those *in vitro* model systems are not specifically orientated toward IE. Different biofilm models with their advantages and limitations have been described intensively elsewhere (Coenye and Nelis, 2010; Lebeaux et al., 2013; Brackman and Coenye, 2015; Roberts et al., 2015; Maske et al., 2017) and will not be included in the present review. They cover both various biofilm physiological, metabolic, genetical behavior, and appearance, as well as methods for preliminary antibiotic susceptibility testing. Considering that the present review is especially focusing on native valve IE, it is also a drawback that most of the biofilm *in vitro* models are based on material adherent biofilms. However, a few specific *in vitro* IE models and an *ex vivo* model have been published (Table 2).

**TABLE 2:** *In vitro* models of infective endocarditis (IE).

Short description of the model	Pathogen	Advantages	Limitations	References
Fibrin clots simulating endocardial vegetations in a pharmacokinetic chamber	*S. aureus, S. mitis* *S. oralis* *E. faecium*	Pharmacokinetics of antibiotics	Lack of other immune components	McGrath et al., 1994; Kang and Rybak, 1995; Hershberger et al., 2000; Vidaillac et al., 2011
	*E. faecalis*	Comparable to results in rabbits		Yim et al., 2017; Kebriaei et al., 2019b Snyder et al., 2014; Werth and Shireman, 2017; Jahanbakhsh et al., 2020
Flow/shear chamber	*S. aureus* *S. lugdunensis* *Streptococci*	Models shear stress related aspects	Artificial	Kerrigan et al., 2008; Claes et al., 2015 Liesenborghs et al., 2016 Tilley et al., 2013; Yakovenko et al., 2018
Pulsatile chamber with native porcine valves	*S. epidermidis*	Models many aspects of a physiological heart	Limited host immune factors	Lauten et al., 2020

*S. aureus, Staphylococcus aureus; S. epidermidis, Staphylococcus epidermidis; S. oralis, Streptococcus oralis; S. lugdunensis, Staphylococcus lugdunensis; E. faecalis, Enterococcus faecalis; E. faecium, Enterococcus faecium.*

One of the first *in vitro* model of IE was performed from *ex vivo* human and canine aortic valves by Gould et al. (1975) to investigate the ability of 14 strains of both Gram-positive cocci and Gram-negative rods to adhere to heart valves and confirmed the superiority of the most frequent IE Gram-positive cocci to adhere to the endothelium compared with the Gram-negative rods. This was followed by an investigation of adherence of *Streptococcus sanguis* to fibrin and platelets in an *in vitro* assay system showing that dextran-producing streptococci increased the ability to adhere to fibrin–platelet matrix. This observation was reconfirmed in the *in vivo* rabbit IE model increasing the probability of IE in the rabbits compared with rabbits inoculated with pretreated bacteria with dextranase (an enzyme that removes dextran from the bacterial cell surface) (Scheld et al., 1978).

#### Simulated Endocardial Vegetations

Later, in 1994, a group around M. J. Rybak (McGrath et al., 1994) published a novel *in vitro* model simulating infective endocarditis (Table 2). They produced human fibrin clots, called simulated endocardial vegetations (SEVs), on monofilament lines by adding *S. aureus* to human cryoprecipitate. A sterile monofilament line was introduced, and bovine thrombin added. The resulting fibrin clot was removed and introduced into ports in a one-compartment model, previously developed for pharmacodynamic studies (Garrison et al., 1990). The model consists of a main chamber that is provided with a flow of fresh medium. Antibiotics or other drugs of interest can be administered directly into the main chamber. To assess the pharmacokinetics and pharmacodynamics of the chosen drugs, McGrath et al. removed the fibrin clots or medium at different points in time and analyzed the bacterial killing and the half-lives of the drugs (McGrath et al., 1994).

Several findings with relevance for IE have been made utilizing this model. The authors have shown that combination treatment of the SEV with humanized doses of different combinations of vancomycin, teicoplanin, and gentamicin could reduce the CFU of methicillin-susceptible *S. aureus* vegetations, while monotreatment with either of the antibiotics had limited effect (McGrath et al., 1994). Those results were comparable with previous observations in rabbit IE models (McGrath et al., 1994). Similar results have been obtained for the combination of vancomycin and quinupristin/dalfopristin against a methicillin-susceptible *S. aureus* (MSSA) and a methicillin-resistant *S. aureus* (MRSA) strain (Kang and Rybak, 1995). While quinupristin/dalfopristin alone was more effective than vancomycin, the combination treatment achieved the highest CFU reductions. At the same time, Kang and Rybak (1995) observed that quinupristin/dalfopristin monotherapy in both bacterial strains led to resistance development toward the compound, as well as to an increase in the erythromycin MIC for the MRSA strain. In the same model, dosing strategies for daptomycin against different MRSA isolates have been evaluated (Vidaillac et al., 2011). By exposing the SEVs to either high (10 mg/kg of body weight/day) or low (6 mg/kg/day) daptomycin doses for 4 days and afterward increasing or decreasing the dose with respect to concentration. The use of high initial antibiotic concentrations followed by a concentration decrease after 4 days was shown to be the most effective strategy. Likewise, combinations with dalbavancin and ceftaroline or telavancin combined with ceftaroline or rifampicin, as well as tedizolid and daptomycin have been tested in the setup against different MRSA or MSSA *S. aureus* isolates (Jahanbakhsh et al., 2018; Smith et al., 2018; Kebriaei et al., 2019a). Additional combinations with daptomycin have been tested against daptomycin non-susceptible MRSA isolates for identification of most optimal antibiotic combinations (Steed et al., 2010). The before-mentioned IE-characteristic inoculum effect was investigated with daptomycin in combination with β-lactams against *E. faecium* predisposed for daptomycin resistance, harboring the LiaFSR substitutions (Kebriaei et al., 2018). Surprisingly, a beneficial effect of combining daptomycin with ampicillin, ceftaroline, or ertapenem was observed (Kebriaei et al., 2018). Daptomycin β-lactam combinations were also tested against the vancomycin-resistant *E. faecium* and *E. faecalis* using SEVs simulating PK/PD, and especially the combination with ceftaroline was promising (Snyder et al., 2014; Werth and Shireman, 2017; Jahanbakhsh et al., 2020).

The effect of daptomycin against *Streptococcus mitis* and *Streptococcus oralis* was evaluated in other recent studies using the SEV model (Yim et al., 2017; Kebriaei et al., 2019b). Here, it was shown that monotreatment with daptomycin was ineffective, with high development of resistance. When daptomycin was combined with ceftriaxone, significant bacterial CFU reductions were observed without formation of daptomycin resistance. On the basis of these observations, the authors suggest further studies on the clinical relevance for the treatment of IE patients (Kebriaei et al., 2019b). The SEV model has also been utilized to study the role of platelets in *S. aureus* IE. The results indicate that platelets can inhibit the bacterial proliferation inside the vegetations dependent on the bacterial susceptibility toward the thrombin-induced platelet microbicidal protein-1 (tPMP) (Mercier et al., 2000).

The SEV *in vitro* model has been validated by comparison with four experimental rabbit models of IE (Hershberger et al., 2000). The study found that the effects of various fluroquinolone treatments against MRSA and MSSA as well as on *E. faecalis* and *E. faecium* were comparable with the results obtained in rabbit vegetations.

#### Shear Flow Model

In an attempt to study the adhesion of bacteria to vessel walls under flow conditions, several groups attempted to model the shear conditions with *in vitro* models. The idea of such flow chambers is to immobilize either bacteria and/or different host components on a surface and then model a flow by leading fluid at the desired flow rate past that surface and the possibility of adjusting the flow rates seen in human vessels.

Such *in vitro* flow models are valuable to identify components involved in the bacterial adhesion to tissues under shear stress. In the context of IE, this can help to understand the process of bacterial adhesion to the endothelial cells during shear stress under the constant flow of fluid.

Using this model, Claes et al. (2015) found that the adhesion of *S. aureus* to endothelial cells under shear conditions was dependent on the presence of von Willebrand factor (vWF) on the endothelium. Interestingly, bacterial adhesion was increased with increasing shear rates (Claes et al., 2015). *In vivo* experiments could confirm the dependence of adhesion on vWF (Claes et al., 2015). The study concludes that shear stress plays an important role in the initial adhesion mediated by vWF of bacteria to the endothelium. Similar observations *in vitro* and *in vivo* have been made for *Staphylococcus lugdunensis* (Liesenborghs et al., 2016). For *S. aureus*, it has also been found that platelet aggregation is dependent on the bacterial clumping factor A and the platelet immunoglobulin receptor FcyRIIa (Kerrigan et al., 2008).

Using a similar *in vitro* technique, Yakovenko et al. (2018) investigated the shear-enhanced binding of streptococci to platelets and found that three streptococcal serine-rich repeat glycoproteins were able to mediate the adhesion to the endothelium under shear conditions. Another publication reports that the glycoproteins Iba and FcyRIIa were important for platelet activation by *Streptococcus oralis* (Tilley et al., 2013). A similar model has also been developed to study the bacterial adhesion to components of graft tissue (Ditkowski et al., 2018).

#### Two-Chamber Compartment Model With Porcine Valves

A recent publication by Lauten et al. (2020) presents a novel IE model, using a pulsatile two-chamber compartment mimicking the heart (Table 2). In this device, the group created monospecies *S. epidermidis* biofilms on native porcine aortic valves under physiologic hemodynamic and temperature conditions. A piston pump establishes pulsatile flow conditions in the chamber that are comparable to the human *in vivo* situation. Bacterial growth in IE can therefore be mimicked taking hydrodynamic factors and physical shear stress into account. The chamber system, as well as the porcine aortic valves were tested to be sterile before inoculation with bacteria. The results indicate that this model can reproduce the pathogenesis and pathophysiology of the biofilm formation on native valves in IE under physiologic flow conditions. However, the model is limited by the lack of host defense factors, such as platelets, fibrin, and leukocytes.

### *In vivo* Infective Endocarditis Model Systems

The history of the experimental model of endocarditis dates to 1878 where Dr. Ottomar Rosenbach was the pioneer to establish the principal of mechanically induced lesions of the heart valves in rabbits (Rosenbach, 1878; Jarcho, 1967) and was followed by a range of other investigators (Jarcho, 1972). The real breakthrough was made in the 1970s with the introduction of polyethylene catheter models, first used in a rabbit right-sided endocarditis model by Garrison and Freeman (Garrison and Freedman, 1970), shortly after, followed by a left-side IE model (Perlman and Freedman, 1971). Durack and Beeson modified the model into streptococcal IE and described the important findings in detail, including the colonization of the sterile lesions, the limited number of leukocytes inside the vegetations, the composition of degraded platelets, and the high grade of bacterial aggregation with low metabolic activity of the deep-seated bacteria compared with the active surface bacteria (Durack and Beeson, 1972a, b; Durack et al., 1973; Durack, 1974). Important experiments of *E. faecalis* IE in rabbits have been performed by E. Gutschik (Gutschik and Christensen, 1978a, b; Gutschik et al., 1979, 1982; Gutschik, 1982). Santoro and Levison (1978) and Kjerulf et al. (1998) reproduced the model in rats, which is now the most used model of experimental IE. Most of the experimental work has been performed on left-sided endocarditis, not only because it is the more clinically relevant for the disease but also due to technical aspects. The *in vivo* model of IE is therefore one of the most investigated infection models known.

#### The Principle of the *in vivo* Model of Infective Endocarditis

Independent of the model animal, the most common way to induce IE is by introducing mechanical damage to the heart valve. The following part briefly describes the most common method for the induction of IE.

**FIGURE 3:**
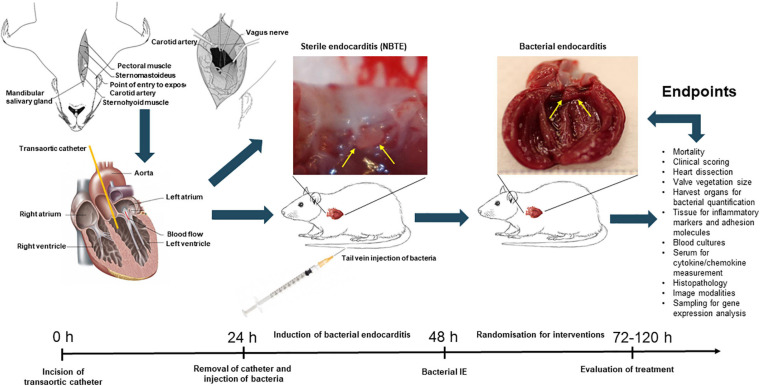
Schematic overview of *in vivo* experimental endocarditis, here illustrated by the experimental model of rat endocarditis. Abbrevations: non-bacterial thrombotic endocarditis, NBTE; infective endocarditis, IE.

**FIGURE 4:**
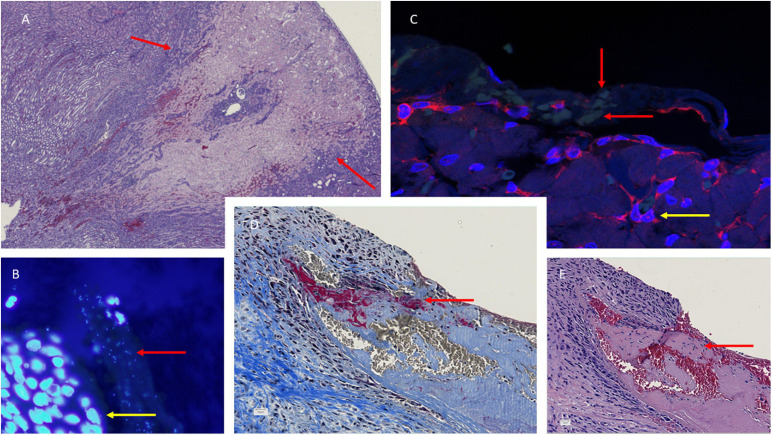
Histology of the aortic valves **(B–E)** and kidney necrosis **(A)** in *S. aureus* infective endocarditis (IE) experimental rat model. Hematoxylin-eosin (HE) staining reveals the large necrosis in the cortex of the kidney in a rat with IE. Representative sections of *S. aureus* infected valve [**(B)**, fluorescens microscope ×100 and **(C)**, confocal microscope, ×62] with DAPI/PNA-flourescence *in situ* hybridization (FISH) stain showing *S. aureus* microcolonies in valve vegetations, red arrow indicating the microcolonies and yellow arrow indicating recruited polymorphonuclear neutrophils. Martius, Scarlet, and Blue (MSB) and hematoxylin-eosin (HE) **(E)** reveals fibrin in red indicated **(D)** and amorphous structures **(E)** in valve vegetations indicated by red arrow, respectively.

The surgery is performed on fully anaesthetized animals (Figure 3). The neck area is disinfected, and a sterile incision of the skin is applied vertically above the sternum. By separating the underlying fascia, the carotid artery is exposed (either left or right). The artery is carefully lifted out and tied off using two sutures. A small hole is introduced into the artery through which a catheter is inserted. The suture toward the heart is removed and the catheter can be pushed down until resistance and pulsation of the catheter is obtained. The catheter is then fixated by caudal suture around the right carotid artery. Excess sutures and catheters are cut, and the skin is closed over the catheter using clips or suture. The catheter can then be left in place for the whole duration of experiments, or it can be removed after 24 h making sufficient mechanical damage to the endothelium of the aortic valves. There are several limitations to leaving the catheter *in situ* during the experiment. First, it increases the mortality and risk of septic and thrombotic embolism; second, it resembles a foreign body infection/IE; and third, it makes long-term observation studies impossible (Kjerulf et al., 1998). After inducing catheter damage of the endothelium of the heart valves, the pathogen of interest is injected in a peripheral vein inducing IE and complications as seen in human IE, here illustrated by aortic valve IE in rats and kidney infarction (Figure 4).

#### Mouse Models of Infective Endocarditis

Even though rabbit and rat models are more prevalent in studying IE, some research has been performed in mice, mostly by induction of endothelial damage on the heart valves through the carotid artery with a catheter (Ring et al., 2014; Gibson et al., 2015). Mice have the advantage over bigger animals to be less expensive, also in housing costs, and that less compounds for treatments are needed. For the induction of endocarditis, microsurgery has to be performed. However, the resulting vegetations cannot be observed macroscopically or isolated individually.

Gibson et al. (2015) developed an MSSA (SA-3529) and MRSA (SA-2015) IE in CD1 mice by inducing aortic valve damage through the left carotid artery. The catheters were kept in place throughout the study duration. The group also attempted an approach through the right carotid artery, which presented more difficulty and was less consistent and was therefore not further used. Bacterial infection was performed by injecting bacteria into the lateral tail vein 18–24 h after placing the catheter. They found that 70% of the mice survive the surgical procedure. Furthermore, they determined the optimal infection rate to be 10E+6 CFU/mouse, as this led to maximal survival compared with higher concentrations, while still showing sufficient infection of the heart. Using histopathology, they confirmed the formation of IE pathology that could be alleviated by vancomycin treatment. They also characterized the time course of their mouse model and found the CFU to increase until 48 h after infection, while more than 80% of the mice did not survive 72 h after infection.

Other groups have contributed to further characterize this mouse model. Ring et al. (2014) performed MRI screening on mouse hearts at 48 h after induction of valve damage *via* the right carotid artery, corresponding to 24 h after the infection with 10E+5 CFU *S. aureus* (ATCC 53657). Their experiments included a sham control group, a group that received unlabeled bacteria, and one group receiving bacteria labeled with iron oxide nano particles. Subsets of the groups receiving labeled or unlabeled bacteria additionally were intravenously injected with an iron oxide nano particle suspension. The iron particles are partly taken up by macrophages and are therefore a way to separate the magnetic resonance imaging (MRI) signal changes resulting from bacteria from the changes resulting from immune infiltration. They achieved comparable surgery survival rates with those of Gibson et al. and found their MRI results to be highly comparable with the standard microbiologically technique, quantitative bacteriology. By comparing the different groups, they confirmed that the changes observed in the MRI resulted from bacterial vegetations and not from macrophage infiltration. Labeling of *S. aureus* was shown to not be required for detection. They concluded that MRI is a promising strategy for precisely and time efficiently monitoring the development of IE in mice; however, the use of MRI to evaluate treatment efficacy remains to be studied. MRI has also been used by other researchers to evaluate a different setup of inducing *S. aureus* IE in mice (Schwarz et al., 2020).

Liesenborghs et al. (2019) compared three different methods of establishing *S. aureus* IE in mice (C57Bl/6). Endocarditis was induced by intravenous injection of bacteria, either in healthy mice (bacteremia group), or by catheter-induced endothelial damage of the heart valves. A new technique applied was a third group, receiving locally infused histamine to activate the endothelium prior to bacterial challenge though a catheter placed at the aortic valves. They found that bacterial adhesion in the bacteremia control group only occurred in mice above the age of 1.5 years, which is a very high age for mice. In the group that received endothelial damage prior to bacterial challenge, the bacterial adhesion was significantly increased compared with the bacteremia group. The highest bacterial adhesion was observed in the histamine-pretreated group.

In a 3-day study, they observed a development of endocarditis in some, but not all, of the mice in the damage-induced model as well as in the histamine-induced model. Their results show that cardiac valve damage, as well as valve inflammation both play a role in the development of endocarditis. Furthermore, they found that the initial bacterial adhesion is mediated by different mechanisms. While *S. aureus* adhesion following heart valve damage is dependent on fibrinogen and Sortase A, the adhesion following cardiac valve inflammation is mediated by platelets.

#### Rat Models of Infective Endocarditis

IE has extensively been studied in rats as described previously. Santoro and Levison (1978) published the first experimental rat IE model of left-sided IE, based on existing rabbit models of right- and left-sided IE. They introduced a polyethylene tubing through the right carotid artery into the left ventricle, leaving it in place for the duration of the experiment. The bacterial challenge was performed 24–48 h after surgery, with 1 ml of overnight cultures of either *Streptococcus mitis*, *S. aureus*, or *E. faecalis*. They could confirm the formation of valve vegetations that were comparable with results obtained from studies performed on rabbits.

Several studies have been performed in experimental rat IE models, which contributed to the understanding of the pathogenesis of the disease and were used for evaluation of numerous therapeutic studies. One study demonstrated that the removal of the catheter before bacterial injection could reduce the efficiency of the infection and that *S. aureus* was superior in establishing IE, compared with *Streptococcus intermedius* and *E. coli* (Heraief et al., 1982). Xiong et al. found that real-time *in vivo* bioluminescent imaging is a valid tool for evaluating antibiotic efficacies (Xiong et al., 2005). However, bioluminescent imaging has limitations because it is less sensitive than conventional bacterial culture, and it might not reveal bacteria, which are less active due to metabolic downregulation (biofilm formation). Furthermore, the stability of the different bioluminescent-modified bacteria varies greatly especially for *S. aureus* compared with other Gram-negative strains. The advances with this method are the reduction in animals needed and the possibility to follow each animal until the evaluation point (Xiong et al., 2005). The plasma and extracellular matrix protein gC1qR/p33 and *S. aureus* clumping factors, as well as coagulase have been found to be important factors in the pathogenesis of *S. aureus* IE (Moreillon et al., 1995; Peerschke et al., 2006). The gene expression of *S. aureus* during IE has been evaluated by Hanses et al. (2014), who found a higher degree of bacteremia and increased vegetation size in diabetic rats compared with non-diabetic rats, underlining diabetes as a risk factor for IE. Microarray analysis revealed an upregulation of 61 *S. aureus* genes, predominantly involved in amino acid and carbohydrate metabolism in the diabetic rats (Smith et al., 2018). *In vivo* gene expression revealed an upregulation of toxins and proteases, although, in general, similar in diabetic compared with non-diabetic rats (Smith et al., 2018).

The experimental model of IE has also been used to investigate the host response of antibody production in rats exposed to the infection (Kjerulf et al., 1998). In this study, Kjerulf et al. observed that a significant specific anti-*E. faecalis* response was not seen until 3 weeks after establishment of the infection, which corresponds to the clinical observation, that antibody measurements are mainly useful in cases of subacute and chronic IE (Kjerulf et al., 1993).

Several experimental rat IE studies have also unraveled new promising treatment strategies for IE. Results from different *in vivo* studies suggest daptomycin and daptomycin combinations with β-lactams as a promising treatment option for recalcitrant *E. faecium* infections (Pericàs et al., 2017; Kebriaei et al., 2018). Recently, animal experiments have suggested that tedizolid is an effective step-down therapy following daptomycin against enterococcal and *S. aureus* endocarditis (Singh et al., 2020).

Other aspects of *in vivo* IE research are focused on finding non-antibiotic adjunctive strategies to supplement antibiotic treatment of IE. Previously, we showed that tobramycin treatment alone was inefficient (Lerche et al., 2015), but could be significantly augmented by supplementing with hyperbaric oxygen therapy (Lerche et al., 2017). We further found the thrombin inhibitor, dabigatran, to be an effective adjunctive treatment strategy to antibiotic (gentamicin) treatment *in vivo* (Lerche et al., 2019) (for details, see the *Antithrombotic treatment in infective endocarditis* section).

#### Rabbit Models of Infective Endocarditis

Experimental rabbit models for IE have been used extensively to understand the disease and find promising new treatment strategies.

Within the last 5 years, the model has mostly been used to investigate bacterial virulence factors and their contribution to the infection of the endocardium and heart valves (Crosby et al., 2016; Herrera et al., 2016, 2017; King et al., 2016; Colomer-Winter et al., 2018a, b; Baker and Nulton, 2019; Reed et al., 2019; Martini et al., 2020). Using rabbit models for IE rather than mice or rats, bears the advantage that although they are still relatively small animals, the rabbit carotid artery is substantially easier to work with due to its size. Furthermore, the immune and cardiovascular system of rabbits is thought to be more comparable with humans (Salgado-Pabón and Schlievert, 2014) and human platelet physiology (Yeaman et al., 1996).

#### Other Species Models of Infective Endocarditis

Apart from the most used models, experimental endocarditis models have also been established in pigs, dogs, and opossums. In pigs, models with (Johnson et al., 1986; Christiansen et al., 2013) and without (Jones, 1972, 1981, 1982) afflicting damage to the heart prior to infection have been published. More recent IE-related research involving pigs is focused on utilizing biological porcine xenografts as valve replacements for elderly patients with aortic valve stenosis and mitral valve regurgitation (Jawad et al., 2020).

Captive opossums have been shown to be susceptible to spontaneously occurring bacteria endocarditis (Sherwood et al., 1968). Sherwood et al. (1971) described an opossum IE model, in which, by giving single i.v. injections of either *Streptococcus viridans* or *S. aureus*, 58% of the *S. viridans* or 100% of the *S. aureus* group of animals developed bacterial endocarditis. Streptococcal endocarditis was mostly located on the left side of the heart, while *S. aureus*-induced endocarditis was seen in both sides of the heart. Injection of three different fungi did not lead to IE induction. Even though the spontaneous development of bacterial endocarditis in opossum points at its usefulness in clinical research, the opossum model has not been used in IE research after 1972. This is mostly due to difficulties in housing and handling the animals, and because the animals were obtained from the wilderness. Also, non-infected opossums in the control groups acquired different infections over the course of experiments.

## Potential Novel Therapies

In the present review, we describe new treatment targets and strategies based on the findings of preclinical studies with the potential to be used in patients with IE.

### Antithrombotic Treatment in Infective Endocarditis

IE is a prothrombic condition and subclinical or silent embolic phenomena that occur in a high number of patients (Hess et al., 2013; Monteiro et al., 2017), especially in *S. aureus* IE as cerebral ischemic strokes (Rasmussen et al., 2009). Embolism is one of the most frequent and serious complications in IE and is primarily related to the size of the vegetations (Røder et al., 1997; Heiro et al., 2000; Di Salvo et al., 2001; Vilacosta et al., 2002; Thuny et al., 2005; Snygg-Martin et al., 2008; García-Cabrera et al., 2013). Since the early antibiotic era, experimental treatment of patients with IE with antithrombotic medication has been tested with unsuccessful outcome (Cooke and Taylor, 1943). Since then, IE treatment with antibiotic and antithrombic therapy has changed significantly. The knowledge of treatment and safety margins for antithrombotic therapy is widely used in the clinical treatment of patients with beneficial outcomes. Later studies have investigated the effect of antiplatelet therapy preclinically, which has shown a beneficial effect (Kupferwasser et al., 1999, 2003).

The basis of anticoagulant treatment of IE dates back to the 1940s where heparin and dicoumarol were used as adjunctive treatment to penicillin therapy (Loewe, 1945; Priest et al., 1946; Thill and Meyer, 1947). In a clinical study from Tornos et al. (1999), examined 56 cases of left-sided native and prosthetic valve *S. aureus* IE in a 12-year period and reported the deleterious effect of antiplatelet therapy with aspirin. Ninety percent (*n* = 21) of patients with prosthetic valve IE receiving anticoagulant treatment displayed a higher mortality due to CNS complications compared with native IE patients. The authors concluded that left-sided prosthetic IE caused by *S. aureus* is a contraindication for anticoagulant treatment.

This study has been the primary reason that the ESC guidelines in 2009 do not recommend anticoagulation (Tornos et al., 1999; Habib et al., 2009). This recommendation was kept in the 2015 guidelines (Habib et al., 2015) based on Chan et al. (2003). However, in a prospective study by Rasmussen et al. (2009), the frequencies of ischemic stroke and intracranial hemorrhage were reported to be 34 and 3%, respectively. Patients who were on anticoagulation therapy prior to diagnosis showed reduced numbers of detectable vegetations, they were smaller, and resulted in an almost fourfold reduction in the number of strokes (OR: 0.27; 95% CI: 0.076–0.96; *p* = 0.04), strongly indicating that anticoagulation reduces the vegetation size. Furthermore, the study did not find any increase in hemorrhagic events in patients receiving anticoagulation therapy, neither in patients with native nor in patients with prosthetic IE. There are several important differences between Tornos’ and Rasmussen’s studies. A much larger number of patients is included in Rasmussen et al. (2009), and the patients were recruited during a considerably shorter time period than in the Tornos et al. (1999) study. The Rasmussen et al. (2009) study also better reflects the current IE management and the higher rate of surgical intervention. However, most studies include prosthetic IE patients already receiving anticoagulation treatment, whereas the majority of patients with native IE in the clinical setting do not receive anticoagulation therapy.

Another prospective study by Snygg–Martin and colleagues studied the role of anticoagulation in 587 episodes of left-sided native IE. The majority of patients were not on anticoagulation treatments, but 8% of the patients received warfarin for indications other than IE. No increase in hemorrhage lesions was seen in the subgroup of anticoagulated patients compared with the group not receiving anticoagulation medicine. Symptomatic cerebrovascular complications were reduced (OR: 0.26; 95% CI: 0.07–0.94) in patients receiving warfarin at admission and were especially prevalent in *S. aureus* IE (Snygg-Martin et al., 2011). A small prospective observational study by Taha et al. (1992) evaluated the effect of low-dose aspirin (75 mg/day) on the incidence of stroke in IE patients and measured the change of the vegetation size by means of echocardiography. Patients receiving aspirin (*n* = 4) did not have embolic complications, while two patients in the control group (*n* = 5) developed focal neurological deficits with cerebral infarction and one of them additionally developed myocardial infarction (Taha et al., 1992).

### Adjunctive Treatment With Dabigatran

Dabigatran is a direct inhibitor of thrombin (FIIa), which is the last serine protease in the clotting cascade converting fibrinogen to fibrin. Thrombin is the most potent platelet activator, further contributing to hemostatic clot formation or thrombus formation in pathologic conditions. Virulence factors of *S. aureus* enhance the clotting cascade and platelet activation leading to a large burst of thrombin and the coagulase-prothrombin complex facilitating the fibrin formation. Dabigatran could therefore be an important treatment strategy by dampening the excess fibrin and clot formation on cardiac valves, resulting in reduced size of the heart valve vegetation and, presumably, a reduced risk of embolization.

Staphylocoagulase is also an important virulence factor and expressed in all *S. aureus* isolates. Coagulase has been known for over 100 years and is important in abscess formation and the pathogenesis of sepsis and IE (Moreillon et al., 1995). Secreted coagulase is central in activation of the coagulation cascade promoting thrombin generation and activating prothrombin without proteolysis (Panizzi et al., 2006). Staphylocoagulase can form a complex with thrombin (Staphylothrombin complex) using fibrinogen as substrate for the conversion of fibrinogen to fibrin (Panizzi et al., 2006) and is important for the expansion of cardiac vegetations (Panizzi et al., 2011). Another coagulase produced by *S. aureus* is vWb protein, also assisting coagulation and clot formation by activation of prothrombin (Kroh et al., 2009). vWb also has adhesion properties resulting in firm adhesion and biofilm formation in valve vegetations (Claes et al., 2014).

Interestingly, it has been shown that dabigatran inhibits the formation of Staphylothrombin complex and reduces the virulence of *S. aureus* in an *in vivo* abscess model in mice (Vanassche et al., 2011). Dabigatran also prevents clotting in a dose-dependent fashion (Vanassche et al., 2010), increasing the time to death in septic mice in combination with anti-ClfA (McAdow et al., 2011), and reduces the fibrin and clot formation (Panizzi et al., 2011; Vanassche et al., 2012, 2013). Furthermore, dabigatran has been effective as a prophylactic treatment in a low-grade *S. aureus* induced model of experimental IE (Veloso et al., 2015).

Several other preclinical studies have also shown dose-dependent beneficial effects of dabigatran by inhibition of the thrombus formation in a venous model of thrombosis (Wienen et al., 2007), and by attenuating inflammation and infarction size in pre-treated rats in a cerebral stroke model (Dittmeier et al., 2016).

These beneficial indications of dabigatran against *S. aureus* led to a recent study by Lerche et al. evaluating dabigatran as adjunctive treatment in an experimental rat model of *S. aureus* IE (Lerche et al., 2019). Here, animals received adjunctive treatment with dabigatran together with gentamicin compared with the control group, receiving gentamicin alone. The dabigatran treated group showed a significantly reduced valve vegetations size compared with the control valves. Furthermore, the bacterial load in valve vegetations was significantly reduced in dabigatran-treated animals, in parallel with key proinflammatory markers of IE (i.e., IL-8 and IL-6). These findings indicate that dabigatran could be a beneficial non-antibiotic treatment option in acute and severe *S. aureus* IE. The authors demonstrated that adjunctive dabigatran treatment reduced the valve vegetation size, platelet aggregation, bacterial load, inflammation, and bacterial dissemination (Lerche et al., 2019). However, these findings need to be evaluated and confirmed in future clinical studies of *S. aureus* IE.

Recently, a single-center, randomized (Peetermans et al., 2018), controlled feasibility, and safety trial of staphylothrombin inhibition with direct thrombin inhibitors in patients with *S. aureus* bacteremia has been performed. Eligible patients were randomized 1:1 to oral dabigatran (110 mg twice a day) or i.v. argatroban for 7–10 days or subcutaneous enoxaparin 40 mg once daily (standard care). Primary outcomes were the feasibility and safety of thrombin inhibitors in patients with *S. aureus* bacteremia. Secondary outcomes included D-dimer evolution (days 0–4) as a marker of coagulation activation, inflammatory and microbiological parameters, and clinical outcomes including metastatic infections. Thirty-one percent (94/303) of screened patients were enrolled. The study showed that similar frequencies of thrombotic and bleeding events occurred in both thromboprophylactic treatment groups, suggesting a similar safety profile of thrombin inhibitors compared with standard thromboprophylaxis based on low molecular weight heparins (LMWH). The secondary outcome parameter D-dimers, which reflects degradation of fibrin, regardless of whether this fibrin is generated by host thrombin or by staphylothrombin, showed a faster resolution in the direct thrombin inhibitor groups compared with the standard LMWH group. In this selected patient group with *S. aureus* bacteremia, direct thrombin inhibitors showed a comparable safety profile to standard care with similar rates of bleeding and thrombotic events.

In a recent Danish nationwide cohort study (Butt et al., 2020), 112,537 patients with atrial fibrillation receiving direct oral anticoagulants were identified from nationwide registries. A median follow-up of 2.0 years was found and 186 patients in the dabigatran group and 356 patients in the factor Xa-inhibitor group were admitted with *S. aureus* bacteremia. The crude incidence rate of *S. aureus* bacteremia was lower in the dabigatran group compared with the factor Xa-inhibitor group {22.8 [95% confidence interval (CI), 19.7–26.3] and 33.8 (95% CI, 30.5–37.6) events per 10,000 person-years, respectively}. In adjusted analyses, dabigatran was associated with a significantly lower incidence of *S. aureus* bacteremia compared with factor Xa-inhibitors (incidence rate ratio, 0.76; 95% CI, 0.63–0.93). These findings, both preclinical and clinical, indicate that adjunctive treatment with dabigatran might have a place in the treatment of *S. aureus* IE. However, as invasive *S. aureus* causes complicated infections with multiple factors influencing the course of disease, future prospective studies in *S. aureus* IE should be made with precautions and selective candidates, with specific relevant end points. A further question is, of course, whether a similar beneficial effect of anti-thrombotic therapy could be expected in IE caused by the other frequent bacterial etiologies since they do not produce coagulase-like molecules.

### Clinical Application of Hyperbaric Oxygen Therapy in Infective Endocarditis

Hyperbaric oxygen therapy (HBOT) is an ancient treatment modality, first documented in 1662 (Henshaw, 1664; Henshaw and Simpson, 1857), where Henshaw built a chamber called “domicilium,” followed by Beddoes in 1795 (Beddoes and Watt) and the first described adjunctive treatment performed by Fontaine (1879).

HBOT is based on the administration of 100% oxygen in a treatment chamber that is pressurized greater than sea level (1 ATA). HBOT enhances the amount of dissolved oxygen in the plasma and increases the supplied oxygen in tissues, independently of hemoglobin (Gill and Bell, 2004). The treatment is commonly applied for 60–90 min at a pressure of 2.5–2.8 ATA corresponding to diving at 15–18 m below sea level. During treatment, the arterial oxygen tension often exceeds 2,000 mmHg (6.8 ml O_2_/100 ml of blood) and levels of 200–400 mmHg in tissues (Thom, 1989). In comparison, the normal oxygen tension in arterial blood is 80–100 mmHg, in venous blood 30–40 mmHg and in a healthy human kidney ∼16 mmHg (Collins et al., 2015). The oxygen concentration in air is 160 mmHg, and in normal human tissue, it is about 60 mmHg (Kanick et al., 2019).

**FIGURE 5:**
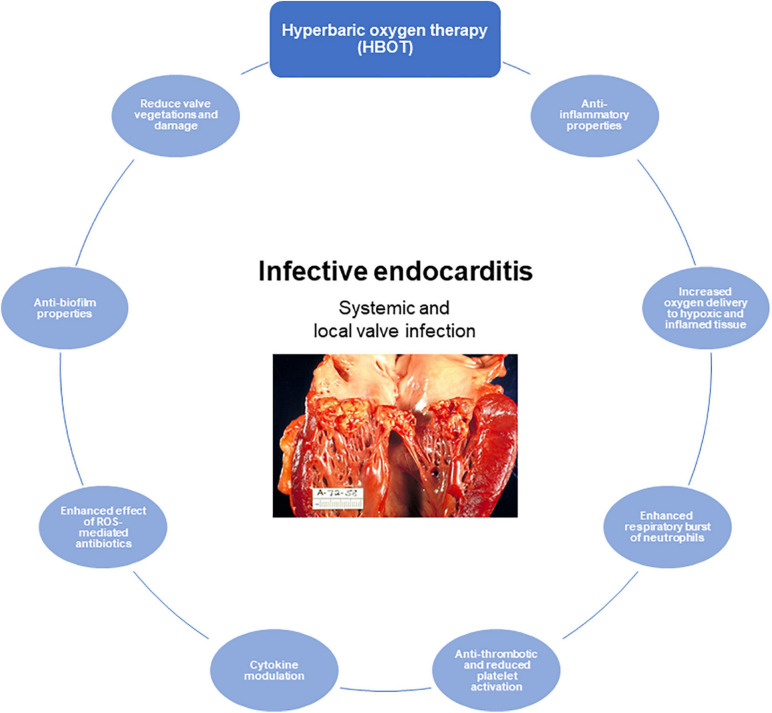
The role of hyperbaric oxygen treatment (HBOT) of infective endocarditis (IE). Proposed and important effects of HBOT in IE. Illustration of human IE reused with permission from Wikimedia Commons, the free media repository.

Hypoxia is a hallmark of infectious diseases, and HBOT is approved and indicated in severe bacterial infections such as necrotizing soft tissue infection (Wilkinson and Doolette, 2004), chronic wounds (Sanford et al., 2018), refractory osteomyelitis (Mader et al., 1978, 1990), and intracranial abscesses (Bilic et al., 2012). Although HBOT is a well-established treatment used both for infectious and non-infectious diseases, there is a need for improved clinical documentation. Hopefully, more randomized control trials are on the way in this area (Hansen et al., 2015). The mechanisms of action of HBOT are numerous and far from all have been completely understood (Figure 5). HBOT potentially affects all cells in the mammalian body due to the fundamental need for oxygen in maintaining cellular functions and survival. In inflammatory diseases, the need for oxygen is increased significantly and HBOT is an important strategy to obtain and maintain cellular function in inflamed hypoxic tissue.

Severe and complicated infections, i.e., *S. aureus* IE, may result in abscess formation and biofilm formation, resulting in low oxygen tension in the inflamed tissue, compromising host responses, and antibiotic effect. *In vitro* studies applying HBOT to treat bacterial biofilms have shown to increase the antimicrobial effect of ciprofloxacin (Kolpen et al., 2016, 2017). Furthermore, by knockout of the catalase gene in *Pseudomonas aeruginosa* (ΔkatA), the oxygen-dependent antibiotic killing is further enhanced (Kolpen et al., 2016). Moreover, HBOT increases the oxygen penetration into the biofilm by a factor of four, increasing the metabolic activity and growth rate of the bacteria, thus, making them more susceptible to antibiotic treatment, within the biofilms (Gade et al., 2018).

A limited number of experimental studies have shown a beneficial effect of HBOT in different *S. aureus* infections (Mader et al., 1978; Turhan et al., 2009; Oguz et al., 2011; Bilic et al., 2012), but in IE, evidence for the benefits of HBOT are sparse (Özkan et al., 2016; Lerche et al., 2017). HBOT might potentially be used as adjunctive treatments for the following reasons: first, to reduce the tissue hypoxia induced by inflammation (Proctor et al., 2014). Second, to enhance the oxygen-dependent killing mechanism by the leukocytes (respiratory burst) (Mader et al., 1978; Jüttner et al., 2003; Almzaiel et al., 2013), decreasing the β_2_ integrin expression of leukocytes thereby reducing the adherence and cytotoxicity (Thom et al., 1997), amelioration of ischemia–reperfusion injury (Thom, 2009), reduce neutrophil extracellular trap (NET)-induced inflammation by neutrophils (Grimberg-Peters et al., 2016), and increase intracellular ROS generation. Third, effects of β-lactam antibiotics, fluoroquinolones, and aminoglycosides are potentiated by oxygen, which leads to the formation of intrabacterial reactive oxygen species, which participate in the toxic killing of bacteria, thereby increasing the antibiotic efficacy (Verklin and Mandell, 1977; Jensen et al., 2014; Kolpen et al., 2016, 2017).

In experimental IE, hyperbaric oxygen has shown similar effects on *S. aureus* biofilms treated with tobramycin and HBOT. Yet, we still need to explore other groups of antibiotics, which are common in the standard treatment of IE, in combination with HBOT Furthermore, we need to evaluate which antibiotics are most efficient together with HBOT for different pathogens. However, two experimental studies have shown augmented effects of HBOT together with linezolid, teicoplanin, and vancomycin in an experimental model of *S. aureus* IE and mediastinitis (Turhan et al., 2009; Özkan et al., 2016).

A preclinical study of adjunctive HBOT in *S. aureus* IE has shown a beneficial effect by improving clinical scores and reducing the bacterial load in the aortic valves, myocardium, and spleen compared with the non-HBOT group (Lerche et al., 2017). Furthermore, the valve vegetation size, weight of the vegetations, and key pro-inflammatory markers was significantly reduced in the HBOT group compared with the control group. These key findings indicate that HBOT has a potential use as adjuvant treatment of IE. These results have also led to the conduction of a clinical feasibility study (currently recruiting) in patients with Gram-positive IE.

Together with the documented positive effects of HBOT in clinical studies of other infectious diseases (Korhonen, 2000; Escobar et al., 2005; Kaur et al., 2012; Skeik et al., 2015; Sanford et al., 2018), this supports the concept of using HBOT in biofilm infections and the rationality of setting up a clinical feasibility study in patients with IE. The benefit of HBOT in IE can most likely be extended to most courses, although the effect in patients that do not respond to conservative antibiotic therapy might be significantly limited. However, IE patients, that are not candidates for invasive cardiac surgery or are in the acute state of IE with severe infections, would probably be likely candidates where HBOT could be highly beneficial.

### Bacteriophage Therapy

With the increase of antibiotic resistance worldwide, there is an increasing interest in developing novel treatments against resistant bacteria. Considering the challenges of antibiotic tolerance of biofilm growing bacteria, some of these novel treatments are also directed against biofilm related infections. Such an approach is the bacteriophage therapy, either alone or in combination with antibiotic drugs. There are limited studies of phage therapy in IE. Oechslin et al. (2017) recently performed an experimental study using a phage cocktail alone or in combination with ciprofloxacin against *Pseudomonas aeruginosa* IE, a very rare pathogen to cause IE. However, the study revealed a 7-log reduction in *P. aeruginosa* in the clots by a single-dose phage (Oechslin et al., 2017). In the rat model, a 2.5-log reduction was observed by both phage and ciprofloxacin monotherapy, whereas the combination revealed a highly synergistic effect with > 6-log killing (Oechslin et al., 2017). *In vitro*, but not *in vivo*, phage-resistant mutants were observed after 24 h, however, this could be prevented by combining the phages with ciprofloxacin (Oechslin et al., 2017). In a case report, anti-*S. aureus* phage therapy with AB-SA01, in a patient with severe *S. aureus* IE on a mechanical aortic valve, native mitral valve, and a possible paravalvular root abscess who could not undergo heart valve surgery, was reported (Gilbey et al., 2019). Although the bacterial strain was antibiotic-susceptible and the patient received treatment with a high dose of flucloxacillin, ciprofloxacin, and rifampicin, blood cultures were repeatedly positive. After the onset of phage therapy, the perpetuating bacteremia vanished, the patient became afebrile, the inflammatory markers were reduced, and the patient recovered after 40 days (Gilbey et al., 2019). Unfortunately, the patient died at day 103 from a new culture negative IE. Although only a case study, the potential of adjunctive bacteriophage therapy in IE remains to be evaluated in randomized controlled trials. The safety of bacteriophage therapy in 13 patients with severe *S. aureus* infection (including IE and septic shock) has been conducted in a single-arm non-comparative trial with no adverse reactions that indicates this as a promising treatment candidate (Petrovic Fabijan et al., 2020). However, bacteriophage therapy as a monotherapy at the current state seems obsolete and should at least be in combination therapy with an efficient antibiotic for a successful outcome (Kebriaei et al., 2021).

### Cyclic Diguanylate(c-di-GMP)[Bis(3′,5′)- Cyclic Diguanyllic Acid]—Disruption of Biofilms

The mechanisms, which lead to biofilm formation per se, are essential for the characteristics and functions of the biofilm-related chronic infections. Therefore, disruption of the biofilm to render it vulnerable to antibiotic treatments or other anti-biofilm strategies have gained much attention. One can argue that the surgical approach is a mechanical disruption of the biofilm, in concordance with the mechanical removal of dental plaques and wound debridement of non-healing wounds. Indeed, uncontrolled infections, in the form of large or enlarging valve vegetations despite appropriate antibiotic therapy, are indications of early heart valve surgery (Habib et al., 2015). A non-surgical manner to disrupt the biofilm structure could be to interfere with the gene regulation of the bacteria during biofilm formation. C-di-GMP is a prokaryotic second messenger implicated in complex biological processes including biofilm formation (Yan et al., 2010; Nair et al., 2017). Adding C-di-GMP to *S. mutans* significantly impaired biofilm formation and tooth surface adhesion, compared with untreated controls (Yan et al., 2010). Likewise C-di-GMP exposure to *S. aureus* has shown reduced intercellular adhesive interaction, as well as reduced biofilm formation and adhesion to the human epithelial cell line HeLa cells (Karaolis et al., 2005). Treatment of mice in a mouse model of biofilm-related mastitis significantly reduced the quantitative bacteriology in a dose-dependent manner (Brouillette et al., 2005). Obviously, this kind of interference with biofilm formation remains to be studied in IE models.

### Antibiotic Combinations

For several biofilm infections, it is well established that combinations of antibiotics, and even combinations of antibiotic drugs with non-antibiotic strategies as discussed above, are essential for optimal outcome (Høiby et al., 2015). Most of these observations have been established in CF and in patients with orthopedic implant infections (Zimmerli and Moser, 2012; Høiby et al., 2015). For antibiotic combinations, *in vitro* experiments have shown how antibiotics affect different niches of the biofilms (Pamp et al., 2008; Herrmann et al., 2010; Siala et al., 2018). Using the flow chamber system and live/dead staining, Pamp and colleagues showed how ciprofloxacin mainly killed active *P. aeruginosa* in the periphery of *in vitro* biofilms, whereas colistin mainly killed the centrally living, but slowly dividing, *P. aeruginosa* (Pamp et al., 2008). A similar finding was obtained combining tobramycin and colistin (Herrmann et al., 2010). For orthopedic implant infections, there are both animal experimental studies as well as clinical observations that show a beneficial effect of adding rifampicin to the antibiotic therapy, if the planktonic pathogens are susceptible (Sendi and Zimmerli, 2017; Zimmerli and Sendi, 2019). Likewise, the addition of rifampicin improves outcome in case of infection on cardiac implants or vascular grafts. For IE on native valves, studies have already stated the beneficial outcome of IE by adding aminoglycoside to β-lactam antibiotics (or glycopeptide) in cases of *E. faecalis* IE or cases with less susceptible streptococci. In the POET trial, shifting to oral antibiotic IE therapy was designed not only as a combination therapy to compensate for variability in pharmacokinetics but also to compensate for biofilm formation on the heart valves (Bundgaard et al., 2019). However, for the remaining cases of IE, it is still debatable whether combination antibiotic therapy is beneficial. If the IE pathogen is highly susceptible to the provided antibiotic drug, and this drug is known to have an acceptable penetration into the vegetation, and the patient is not critically ill, antibiotic monotherapy in correct dosages is probably sufficient. Although, as mentioned previously, resistant SCVs may be selected. However, in cases of prostheses, large vegetations, abscess formation, recurrent infection, enlargement of vegetation, unsecure pharmacokinetic, or critical illness, using two bactericidal antibiotic drugs with different microbe killing mechanisms in combination is a recommended strategy in our opinion.

## Conclusion

In the present review, we describe how the observed characteristics of some of the most prevalent and important pathogens causing IE, meet and fulfill the criteria for IE being considered a biofilm infection. Within the vegetations, microorganisms present as biofilm-like aggregates that are responsible for the recurrent and chronic nature of biofilm infections. They show the typical extensive tolerance of biofilms to antibiotic therapy and the host response.

In all cases, IE is a serious fatal infection if not treated. Even following the guidelines for IE treatment, there is a high mortality rate of 20–25%, and a 1-year mortality rate of up to 40% in some reports. However, this represents a wide spectrum of courses of left-sided IE from relatively mild cases responding very well to a 2-week antibiotic course using the IE dosages (and combinations), to serious infections with septic shock, heart valve destruction, and severe embolization. This calls for an optimization of therapy to improve the clinical outcome. Accepting IE as a biofilm infection can be an inspiration for novel treatment candidates, as elaborated in the present review.

One can argue that if early infection control is achieved in IE, there is a substantial likelihood of treatment success. Therefore, in contrast to the chronic pulmonary biofilm infection in CF and orthopedic alloplastic-related infections, where it is almost impossible to eliminate an already established infection. IE is a curable biofilm infection for several reasons (Høiby et al., 2015). It is important to recognize that almost 50% of all IE patients in specialized referral centers and approximately one-third are undergoing valve replacement surgery for various indications, one of them being lack of infection control (Iversen et al., 2019). Additional indications include abscess formation, extended valve destruction leading to heart failure, and embolization leaving a rest vegetation. The beneficial outcome of conservative antibiotic treatments may also be due to the location of the infection, centrally in the blood stream with optimal antibiotic concentrations. It probably also can be of significance that the valves are living tissue, in contrast to prostheses.

In IE, model systems are essential, since it is difficult to perform controlled and randomized clinical trials. However, in the present review, we have covered the major model systems available for research in IE. From the observations, it can be seen that the *in vitro* models, although helpful, may not sufficiently mirror the ongoing IE in the patients. The animal models are all reflective of the clinical IE when focusing on the valve pathology and suitable for initial investigation of new treatment strategies, both for antibiotic as non-antibiotics. However, animal models are technically difficult and may not lead to the clinical impact they may deserve—since there is a substantial gap to the clinical trials. However, preclinical studies are important for testing hypotheses that could be evaluated in clinical trials.

## Author Contributions

CL and FS contributed equally to the literature collection and the writing of the manuscript. MT, EF, KI, and HB edited and commented on the final manuscript. NH contributed with invaluable knowledge and comments to the manuscript and edited the final manuscript. CM was the senior and corresponding author, wrote the draft, provided the ideas, supervised the entire process from inception to the final submission, and edited the final manuscript. All authors contributed to the article and approved the submitted version.

## Conflict of Interest

The authors declare that the research was conducted in the absence of any commercial or financial relationships that could be construed as a potential conflict of interest.
